# Loss of ‘Blue Carbon’ from Coastal Salt Marshes Following Habitat Disturbance

**DOI:** 10.1371/journal.pone.0069244

**Published:** 2013-07-08

**Authors:** Peter I. Macreadie, A. Randall Hughes, David L. Kimbro

**Affiliations:** 1 Plant Functional Biology and Climate Change Cluster (C3), School of the Environment, University of Technology, Sydney (UTS), New South Wales, Australia; 2 Marine Science Center, Northeastern University (NU), Boston, Massachusetts, United States of America

## Abstract

Increased recognition of the global importance of salt marshes as ‘blue carbon’ (C) sinks has led to concern that salt marshes could release large amounts of stored C into the atmosphere (as CO_2_) if they continue undergoing disturbance, thereby accelerating climate change. Empirical evidence of C release following salt marsh habitat loss due to disturbance is rare, yet such information is essential for inclusion of salt marshes in greenhouse gas emission reduction and offset schemes. Here we investigated the stability of salt marsh ( *Spartina* *alterniflora* ) sediment C levels following seagrass ( *Thallasiatestudinum* ) wrack accumulation; a form of disturbance common throughout the world that removes large areas of plant biomass in salt marshes. At our study site (St Joseph Bay, Florida, USA), we recorded 296 patches (7.5 ± 2.3 m^2^ mean area ± SE) of vegetation loss (aged 3-12 months) in a salt marsh meadow the size of a soccer field (7 275 m^2^). Within these disturbed patches, levels of organic C in the subsurface zone (1-5 cm depth) were ~30% lower than the surrounding undisturbed meadow. Subsequent analyses showed that the decline in subsurface C levels in disturbed patches was due to loss of below-ground plant (salt marsh) biomass, which otherwise forms the main component of the long-term ‘refractory’ C stock. We conclude that disturbance to salt marsh habitat due to wrack accumulation can cause significant release of below-ground C; which could shift salt marshes from C *sinks* to C *sources*, depending on the intensity and scale of disturbance. This mechanism of C release is likely to increase in the future due to sea level rise; which could increase wrack production due to increasing storminess, and will facilitate delivery of wrack into salt marsh zones due to higher and more frequent inundation.

## Introduction

Salt marshes are one of the most powerful carbon (C) sinks on the planet. They bury at a rate ~55 times faster than tropical rainforests (regarded as one of the most significant terrestrial C sinks), and their global carbon burial (up to 87.2 ± 9.6 Tg C yr^-1^ based on preliminary assessments) appears to exceed that of tropical rainforests (53 ± 9.6 Tg C yr^-1^). These rates are particularly staggering given that salt marshes occupy only a small fraction (0.1-2%) of the total land area of tropical rainforests [1]. Furthermore, salt marshes can store C for millennia [2,3], whereas rainforests usually only store C for decades. Despite their value as C sinks, salt marshes have undergone rapid global decline [25% since the 1800s; 4,5], particularly due to landscape conversion for housing and farming [6,7]. This raises concerns that society is losing an important C sink, and that large amounts of ancient buried C are being released into the atmosphere as CO_2_ and contributing to global warming.

Disturbance is likely to affect the C sink capacity of salt marshes in four main ways. First, salt marshes filter and capture laterally-imported allochthonous C that contributes to the below-ground sediment C stock; therefore, loss of salt marsh plant material following disturbance could reduce this particulate C trapping capacity, thereby causing a reduction in C stock accumulation [8,9]. Second, disturbance of salt marshes could reduce the overall plant biomass contributing to C capture via photosynthesis [10]; this loss of photosynthetic capacity could also reduce the total amount of C captured by salt marshes [11]. Third, disturbance of salt marsh could cause loss of C stored in plant material itself (structural C) due to plant die off [12], which could be exported and lost from salt marsh ecosystems if it does not make its way into the salt marsh sediment C stock [13]. Fourth, and perhaps most importantly, disturbance of salt marsh could result in the release of buried ancient sedimentary C via erosion, leaching, and microbial mineralization [14].

There is relatively little empirical evidence for C loss from salt marshes following disturbance, yet such information is essential for inclusion of salt marshes in greenhouse gas emission reduction and offset schemes (e.g. the United Nation’s programme on Reducing Emissions from Deforestation and Forest Degradation - UN-REDD+). Most studies that have reported loss of C from salt marshes have been done in systems that have been broadly classified as ‘wetlands’. Although these may include salt marsh, they typically consist of mixed habitats, complicating attempts to ascertain the responses of salt marsh responses *per se*. Furthermore, these studies are usually based on landscape-scale conversions (e.g. conversion into farmland), and there appears to be no data on the effects of smaller-scale (within-habitat) disturbances, which are increasingly common and ecologically relevant [15,16,17]. Nevertheless, we do know that landscape-scale disturbances of wetlands can cause significant depletion of organic C (e.g. 96% [18]) and weakening of the C sequestration capacity [2,19].

**Figure 1:**
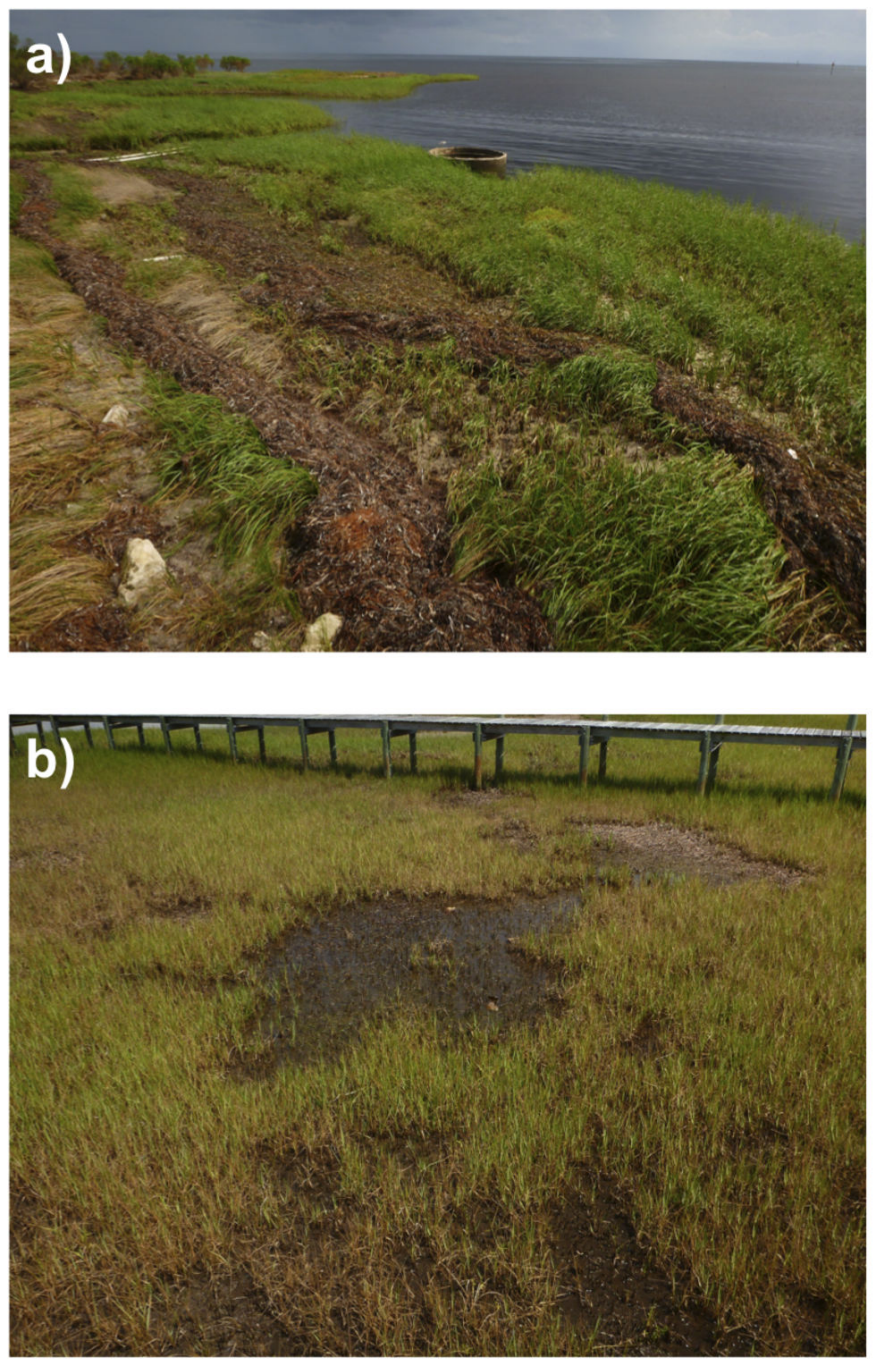
Photographs showing disturbance to salt marsh (* Spartina alterniflora *) caused by accumulation of seagrass wrack. A) fresh wrack (* Thallasia testudinum *) accumulation after a storm; and B) a ‘halo’ of bare sediment after prolonged wrack smothering.

The purpose of this study was to quantify the effects of small-scale (within-habitat) disturbance and concomitant habitat loss on the C sink capacity of salt marsh. Specifically, through analyses of sediment cores, we tested the hypothesis that disturbed areas of salt marsh dominated by the marsh grass *Spartina* *alterniflora* (hereafter referred to as ‘salt marsh’) would have significantly lower amounts of organic C than adjacent undisturbed salt marsh. We then explored which C pools (below-ground plant C biomass vs. sedimentary organic C) are vulnerable to disturbance, and the ecological consequences of these losses. In our study, we capitilised on a natural disturbance event that is common along the Gulf Coast of the USA (and elsewhere around the world): die-off of salt marsh due to seagrass ( *Thalassia* *testudinum* ) wrack accumulation, which occurs when senesced or storm-fragmented seagrass leaves are deposited in the marsh at high tide; when this wrack remains in place for extended periods of time, it smothers the underlying salt marsh plants and causes localised die off (Figure 1).

## Methods

### Site and experimental design

This experiment took place in St Joseph Bay, Florida (29° 41'13.87''N, 85° 19'43.67''W) on private land (permission granted by L. Hughes). The experiment did not involve endangered or protected species. At this site, we observed salt marsh disturbance and habitat loss due to wrack accumulation, which had caused patches of bare sediment in otherwise continuous salt marsh meadows (Figure 1) that had persisted for 3-12 months prior to our sampling. Within our study site (total area 7,275 m^2^) we counted and measured the area of each patch using aerial images. This was achieved using Google, Earth’s polygon function to mark out the perimeter of each halo (imagery date 1/3/2012), followed by Earth Point (www.earthpoint.us) to measure the area of each halo polygon. We haphazardly selected 10 patches, and measured the amount of organic carbon in these bare areas, which we called ‘disturbed’, and compared this to adjacent ‘undisturbed’ salt marsh plots at an equivalent tidal height. Each bare, disturbed area typically represents a separate wrack mat, and we therefore treated each disturbed-undisturbed plot pair (n = 10) as independent.

### Survey 1: Do below-ground C pools decrease following salt marsh loss?

This first survey was conducted to compare: (1) overall differences in C stocks between disturbed patches and the surrounding undisturbed meadow; and then (2) to compare organic C levels at different depths down the core. Sediment cores were extracted via a piston corer, which involved hammering a PVC tube (150 cm length, 4 cm internal diameter) into the sediment, and using suction from the tightened piston located within the tube to hold the sediment in place while the tube was extracted. Once extracted, the sediment in the tube was extruded *in situ* and divided into the following sections: 0-1, 1-3, 3-5, 5-10, and 10-15 cm (note: analyses were restricted to the upper 15 cm since we were only interested in changes relevant to recent disturbance). These sediment samples were then placed into sterile sample bags (Nasco Whirl-Pak®) on ice, and then transported back to the laboratory for analysis of C content.

### Survey 2: Are declines C levels in disturbed plots due to loss of plant biomass?

We performed a second survey to determine whether subsurface losses of C identified in the first survey were due to loss of plant biomass. This second survey involved re-sampling disturbed and undisturbed plots (n = 5 pairs) at the same study site so that we could separate plant (structural) C from sedimentary C (in Survey 1 they were pooled). The procedure involved the same coring, extrusion, sectioning, transportation, and sample analysis procedures (i.e. LOI) as described in Survey 1, with the exception being that the core sections were rinsed through a 2mm sieve using de-ionized water to separate plant biomass from the sediment, thereby allowing the plant and sediment fractions to be analysed for C content separately.

### Sediment C content analyses: Loss on ignition

To enable C stock to be calculated, and to ensure that there were no differences in porosity among disturbed and undisturbed sediment samples, we calculated dry bulk density (g cm^-3^) for each sediment depth by dividing the mass of the dried sediment by the original (pre-dried) volume of the sample. We then used standard loss on ignition (LOI) procedures to determine organic C concentrations; Craft et al. [20] has shown LOI methods to be an excellent predictor of organic C concentrations in salt marshes. The LOI procedure involved drying at 105^°^C for 3 hours to obtain weight, followed by combustion at 525^°^C for 3 hours to obtain combusted weight. At 525^°^C, acidification of samples is not necessary because only the organic (not inorganic) C is lost (i.e. volatilised). Furthermore, pH levels (6.4 ± 0.19 [21]) in the porewater of *S* *. alterniflora* salt marsh sediments in this region are generally too low to support carbonate (inorganic) precipitation, and shell-forming organisms were rare at our study site.

Following Craft et al. [20], the following quadratic equation was used to convert LOI values into organic C concentrations: Organic C = 0.40(LOI) + (0.025 x LOI)^2^. C stock (i.e. soil carbon per hectare; Mg ha^-1^) was determined by summing the soil C mass for each depth interval: soil C (Mg ha^-1^) = bulk density (g cm^-3^) x soil depth interval (cm) x % C. As noted earlier, we limited the scope of our investigation to upper (0-15 cm) zone. Therefore, our data should only be used to compare overall differences in C stock between disturbed and undisturbed salt marsh plots, and not for estimating the size of entire soil carbon pool – the latter would require much deeper cores; 1 m minimum (as is convention in many C accounting programs), or, ideally, down to the bedrock.

### Statistical analyses

To understand the results of our first survey, we used a model-selection approach that identified the most parsimonious explanation of the overall C stock and then organic C levels at different depths. Model selection offers an alternative approach to traditional null hypothesis testing, whereby several competing hypotheses are simultaneously confronted with data to allow identification of a single best model that lends support to a particular hypothesis [22]. Although relatively new to the field of landscape ecology, model selection is widely accepted and well-developed in other fields (e.g. molecular systematics [22]).

In this study, for each response variable, the model selection procedure involved creating a series of nested linear models that ranged from simple to complex. The simplest model represented (a) the null model with an intercept of 1. While analysis of the C stock results only required the further consideration of a single-factor model that distinguished between plot type (i.e., disturbed vs. undisturbed), analysis of the organic C data required us to consider (b) all possible single-factor models based on sediment depth and plot type (disturbed or undisturbed), (c) a two-factor additive model with sediment depth and plot type, and (d) a two-factor model with an interaction term between sediment depth and plot type. After constructing these nested models, we used AIC_c_ (Akaike Information Criteria, with a correction factor for small sample size) to identify the most parsimonious model [23]. Model rankings were based on weight scores (*w*_*i*_), which were calculated as the model likelihood normalized by the sum of all model likelihoods; values close to 1 indicate greater confidence in the selection of the best model (Burnham and Anderson 1998). We also calculated the difference between AICc of a particular model and the AICc of the most likely model (i.e., model with *w*_*i*_ closest to 1). If this observed difference (or ∆AIC score) between the top candidate model and the alternative model under consideration was greater than 2.0, then we considered the top candidate model significantly stronger [24].

For the analysis of results from the second survey, we repeated the latter model-selection approach. In all models, plot number (consisting of paired disturbed and undisturbed plots) was included as a random effect. Candidate models and their ∆AIC scores and AIC weights are provided in tables. Analyses were conducted with R statistical software (version 2.11.1) using the lmer function in the lme4 package and the AICctab function in the bblme package [23].

## Results

**Figure 2:**
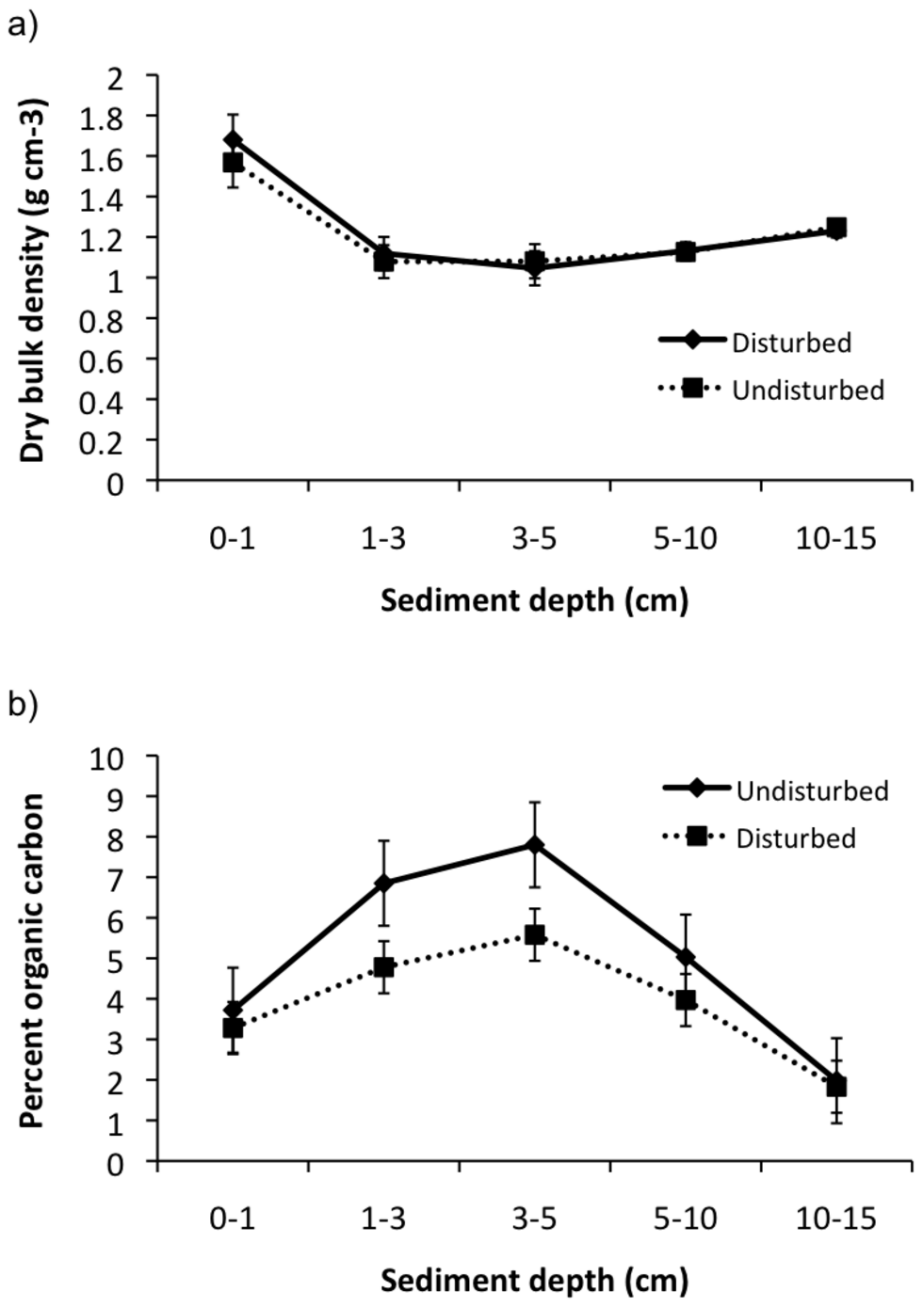
Organic carbon content (mean ± standard error) of sediment at different depths below the surface. Samples taken from disturbed and undisturbed salt marsh (* Spartina alterniflora *).

We recorded a total of 296 disturbed patches within our study site, which contained an otherwise continuous salt marsh meadow of area 7,275 m^2^ (equivalent to the area of a soccer field). The average area of these disturbances was 7.5 ± 2.3 m^2^ (mean m^2^ ± SE). C stocks in the top 0-15 cm were 23.76 ± 1.02 Mg C ha^-1^ in disturbed plots and 29.81 ± 1.76 Mg C ha^-1^ in undisturbed plots. Dry bulk density was consistent in both disturbed and undisturbed plots (Figure 2a), showing a slight (~30%) decline from 0–1 cm to 1-3 cm, but then remaining relatively constant throughout the rest of the core. Sediment organic C content in disturbed and undisturbed areas of salt marsh both showed a similar ‘hump’ shaped distribution in their organic C content with depth (0–15 cm, Figure 2b), reaching a peak in the 3–5 cm zone (undisturbed - 5.1 ± 1.0%; disturbed 3.9 ± 0.6%; mean ± SE). Sediment organic C content was best explained by an interaction between plot type (disturbed vs. undisturbed) and sediment depth (Figure 2b Table 1): undisturbed plots had higher sediment organic C than disturbed plots, but only at depths of 1–5 cm (Table 1).

**Table 1:** Results of nested linear mixed-effect models for the relationship between salt marsh (* Spartina alterniflora *) plot type (disturbed or undisturbed), sediment depth, and sediment organic carbon (C) in natural marshes.

Response variable	Model	df	dAIC	Weight
Sediment C	C = Intercept + (Plot)	3	21.1	<0.001
	C = Plot type + (Plot)	4	19.8	<0.001
	C = Depth + (Plot)	7	5.6	0.052
	C = Plot type + Depth + (Plot)	8	4.3	0.100
	C = Plot type * Depth + (Plot)	12	0.0	0.848
Sediment C at depths of 1-5 cm	C = Intercept + (Plot)	3	2.9	0.189
	**C = Plot type + (Plot)**	4	0.0	0.811

The plot number (representing one disturbed and one undisturbed paired plot) was included as a random effect. Bold indicates best model. Parentheses denote random effect. dAIC is the difference between the AICc of a particular model compared to the lowest AICc observed. If dAIC is less than 2.0, models are considered equivalent. The Akaike weight is calculated as the model likelihood normalized by the sum of all model likelihoods; values close to 1.0 indicate greater confidence in the selection of a model. The intercept in the null model is 1.0.

**Figure 3:**
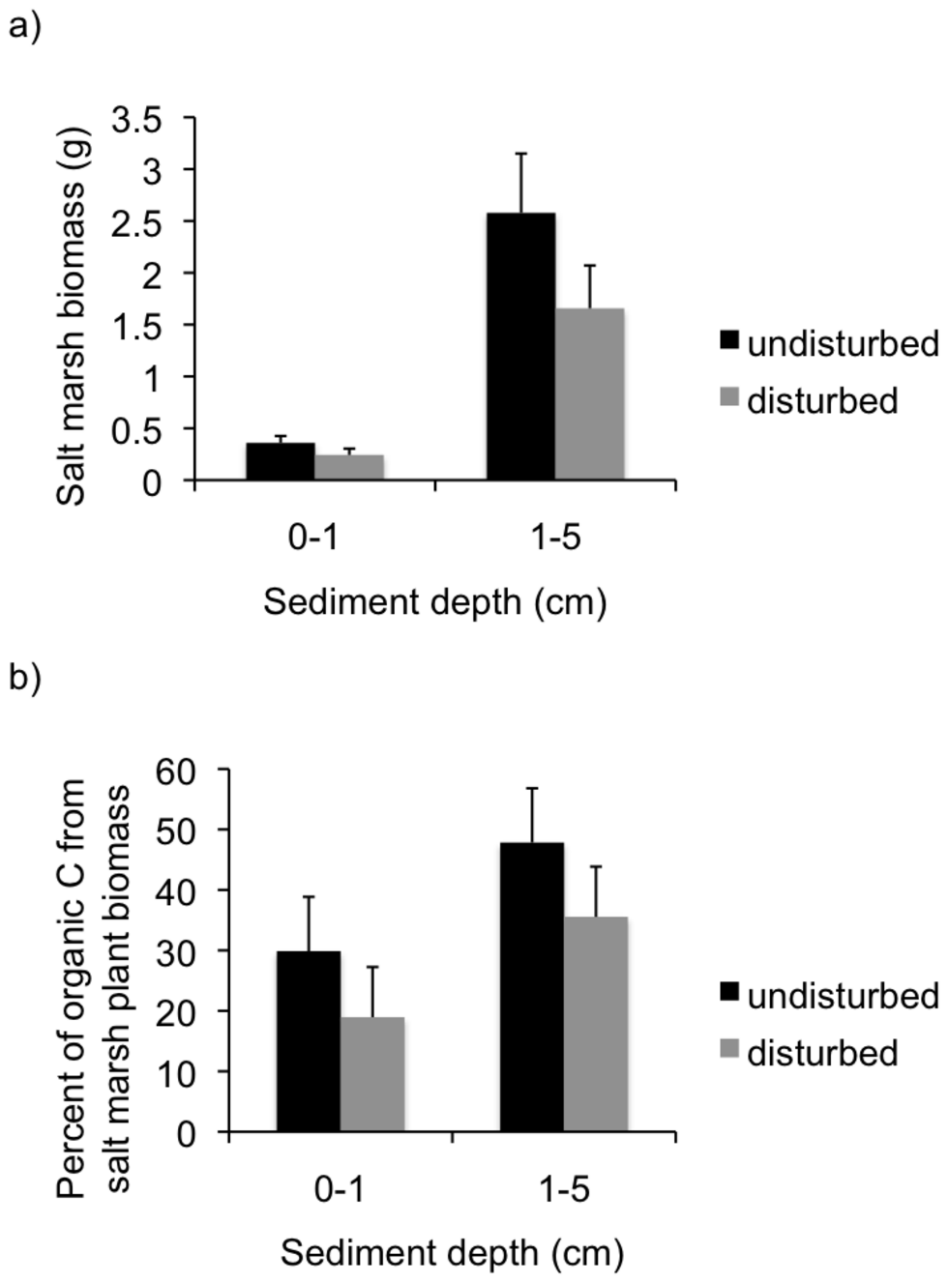
The amount of salt marsh plant material (mean + standard error) in sediments from disturbed and undisturbed plots. Sediment cores were sectioned into 2 depths and all plant material was quantified to determine its contribution to the organic carbon content of the sediments. a) Absolute biomass of salt marsh ( *Spartina* *alterniflora* ) in sediment cores from disturbed and undisturbed plots at two depth intervals. Plant biomass was higher in the 1-5cm depths than in the surface sediments. b) Percentage of organic carbon (C) in each sediment core resulting from salt marsh plant biomass in disturbed and undisturbed salt marsh sediments.

Because this depth region corresponds with the salt marsh rhizome and rooting zone ( [25]; R. Hughes, unpublished data), we hypothesized that loss of salt marsh biomass (root material) in the undisturbed plots caused this difference. Our subsequent sampling was consistent with this expectation: the biomass of salt marsh root material lost on ignition was much higher in the 1–5 cm depth range than in the surface sediments (Figure 3a). In addition, there was equal support for the candidate model with an additive effect of plot type and depth (∆AIC score 1.6), with higher salt marsh biomass in undisturbed than disturbed plots (Figure 3a Table 2). Taken together, these two models are needed to best explain our results (i.e., combined w = 0.90 [22]). Similarly, the percentage of organic carbon in the sediments that came from salt marsh was best explained by a candidate model that only distinguished between plot type and a two-factor additive model that distinguished between plot type and depth (combined *w* = 0.99; Figure 3b Table 2).

**Table 2:** Results of nested linear mixed-effect models for the relationship between plot type (disturbed or undisturbed), sediment depth, and the amount of salt marsh (* Spartina alterniflora *) organic carbon (C) in natural marsh sediments.

Response variable	Model	df	dAIC	Weight
Salt marsh C (g)	Salt marsh C = Intercept + (Plot)	3	13.0	<0.001
	Salt marsh C = Plot type + (Plot)	4	14.7	<0.001
	**Salt marsh C = Depth + (Plot)**	**4**	**0.0**	**0.605**
	**Salt marsh C = Plot type + Depth + (Plot)**	**5**	**1.6**	**0.278**
	Salt marsh C = Plot type * Depth + (Plot)	6	3.3	0.115
Salt marsh C (%)	Percent salt marsh C = Intercept + (Plot)	3	22.1	<0.001
	Percent salt marsh C = Plot type + (Plot)	4	16.1	<0.001
	Percent salt marsh C = Depth + (Plot)	4	10.3	0.004
	**% Salt marsh C = Plot type + Depth + (Plot)**	5	1.8	0.290
	% Salt marsh C = Plot type * Depth + (Plot)	6	0.0	0.706

The plot number (representing one disturbed and one undisturbed paired plot) was included as a random effect. Bold indicates best model(s). Parentheses denote random effect. dAIC is the difference between the AICc of a particular model compared to the lowest AICc observed. If dAIC is less than 2.0, models are considered equivalent. The Akaike weight is calculated as the model likelihood normalized by the sum of all model likelihoods; values close to 1.0 indicate greater confidence in the selection of a model. The intercept in the null model is 1.0.

## Discussion

Our data shows that wrack-induced disturbance causes a decline in organic C levels in salt marsh sediments. This loss was evident in the upper subsurface samples (1-5 cm depth zone), wherein disturbed salt marshes showed significantly lower (~30%) amounts of organic C than undisturbed salt marsh. Additional sampling indicated that this reduction in organic C in disturbed salt marsh was due to loss of belowground plant biomass; an otherwise important component of the C stock [26]. This amount and rate of belowground biomass loss is consistent with litterbag data by Benner et al. [27], who reported 55% loss of *S* *. alterniflora* belowground biomass after 18 months. As for the lack of detectable loss of C from the top surface (0-1 cm), which is a more dynamic zone in terms of C movement, the data suggests that this zone may have been subsidised by C from the seagrass wrack.

**Figure 4:**
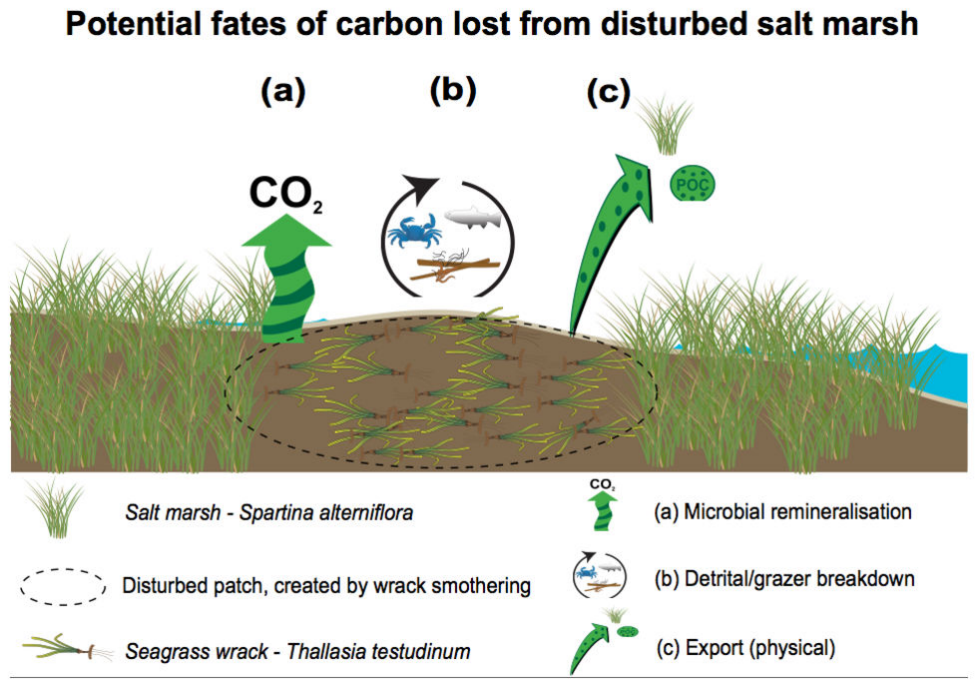
Conceptual diagram illustrating the possible fates of carbon (C) lost from disturbed salt marsh: (a) remineralization of C by microbes and release as CO_2_; (b) consumption of C by detritivores and grazing animals; (c) physical export (into deeper water, other parts of the marsh, or other habitats), which could lead to re-burial of the C. Diagram produced using the Integration and Application Network (IAN), University of Maryland Center for Environmental Science, Cambridge, Maryland.

Is the loss of C from disturbed salt marsh in this study ecologically meaningful? Both the amount of habitat loss caused by wrack disturbance (~30% of the total habitat area) and the amount of C loss from disturbed areas (30% from the subsurface zone; 1-5 cm) is high, but unless the C released during disturbance ends up as atmospheric CO_2_, there may be little direct negative impact on the environment as far as climate change is concerned. Possible fates of the lost C include (Figure 4): (1) physical export (into deeper water, other parts of the marsh, or other habitats), which could lead to re-burial of the C; (2) consumption by grazing animals; or (3) mineralization by microbes and release as CO_2_. Relatively little is known about the transformation fate of belowground biomass [27], but Davis et al. [28] showed that microbes play a major role and abiotic forces interact to control CO_2_, even in the anoxic zone. Their research suggests that microbes actually help to keep C buried within sediments under normal conditions, but that physical disturbances can trigger abiotic release of C, and can also prime microbes into metabolising buried C. Further work (e.g. C tracing experiments) is needed to determine the fate of the 30% subsurface loss of C from our disturbed plots.

We feel that it is important to point out the potential fates of lost C stock given loss of C stock from ‘blue carbon’ habitats (e.g. seagrasses, salt marshes, and mangroves) is often assumed to constitute evidence that these habitats are releasing CO_2_ into the atmosphere. Pendleton et al. [29] estimated that, given current rates of tidal marsh conversion (1-2% yr^-1^), total global C emissions is likely to be 0.02-0.24 Pg CO_2_ yr^-1^, which equates to an economic loss of 0.64-9.7 billion US$ yr^-1^ if CO_2_ is priced at US$41 per ton. In addition to assuming that lost C stock from marshes is released as atmospheric CO_2_, this figure also assumes that the entire top meter of C in sediment and plant biomass is released from marshes following habitat loss. There is little information on which pools (depths) of C are vulnerable to release. Certainly, without the aboveground plant material, surface layers of C are more vulnerable to efflux due to physical forces (e.g. waves, currents), but it would require substantial changes in hydrodynamics to remove the upper metre of C.

Regardless, of whether the C lost from disturbed salt marsh contributes to atmospheric CO_2_, there would certainly be weakening of the C sink capacity of the salt marsh due to loss of trapping capacity of particulate C, which contributes to the sediment C pool [30], although this is likely to take years to become detectable from C stock measurements. This lack of trapping capacity, along with the lack of plant material contributing towards the belowground C pool, would mean that salt marshes would be unable to accrete at the same rate as undisturbed areas of salt marsh [31]. Furthermore, without the aboveground plant material to buffer against hydrodynamics forces that cause erosion, there is potential for subsidence of disturbed salt marshes. Therefore, even though loss of C from disturbed marshes does not constitute evidence that disturbance to salt marshes contributes to rising atmospheric CO_2_ levels, there is certainly a strong rationale that disturbance weakens the C sink capacity of salt marshes.

Given the high level of current interest in the value of salt marshes as blue C sinks [1,32], we recommend that further research is needed to develop accurate C budgets in disturbed and undisturbed salt marshes. These C budgets need to consider all possible fates of C [33], as well as the influence of environmental conditions (incl. extreme weather events and future climate change) on those possible fates. The latter should include investigation of the relative importance of biotic (e.g. microbial processes, bioturbation) versus abiotic (e.g. physical forces that can trigger CO_2_ efflux) processes in facilitating C loss from salt marsh following disturbance. Furthermore, longer-term studies (years - decades) are needed to determine whether deeply buried (>1 m) C stocks are vulnerable to being depleted from disturbed salt marshes. Finally, there is need to understand the potential for habitat rehabilitation to restore C stocks in disturbed salt marsh ecosystems [34]. Recent technological advances – in particular, eddy covariance techniques for measuring ecosystem-level carbon exchanges [35] – will make the task of developing C budgets in salt marshes much easier.

## Conclusion

In conclusion, our study provides evidence that localized disturbance to salt marshes can cause loss of buried C, which has important implications for nature-based climate change mitigation programs if this C is released into the atmosphere as CO_2_ (as opposed to being re-buried). Disturbance and concomitant loss of salt marsh habitat via wrack accumulation is understudied [36], yet it could be one of the major causes of C stock loss in salt marshes. Wrack accumulation is a natural process, and there is little that could be done to prevent it occurring; however, it is important to understand how this process of disturbance is likely to affect C budgets, and whether restoration is necessary to stop C release, especially since the frequency and intensity of this disturbance is predicted to increase with future sea-level rise [36], which will increase both wrack production and salt marsh inundation [37].
